# Imaging, Tracking and Computational Analyses of Virus Entry and Egress with the Cytoskeleton

**DOI:** 10.3390/v10040166

**Published:** 2018-03-31

**Authors:** I-Hsuan Wang, Christoph J. Burckhardt, Artur Yakimovich, Urs F. Greber

**Affiliations:** 1 Division of Virology, Institute of Medical Science, the University of Tokyo, Tokyo 108-8639, Japan; jessica.ihwang84@gmail.com; 2 Lyda Hill Department of Bioinformatics, UT Southwestern Medical Center, Dallas, TX 75390, USA; Christoph.Burckhardt@utsouthwestern.edu; 3 MRC Laboratory for Molecular Cell Biology, University College London, London WC1E 6BT, UK; a.yakimovich@ucl.ac.uk; 4 Department of Molecular Life Sciences, University of Zurich, Winterthurerstrasse 190, CH-8057 Zurich, Switzerland

**Keywords:** Modeling, simulation, computing, quantitative microscopy, fluorescent virions, microscopy, single particle tracking, trajectory segmentation, click chemistry, tracking, trafficking, membrane traffic, fluorescence microscopy, immunofluorescence microscopy, electron microscopy, microtubule, intracellular transport, machine learning, virus infection mechanisms, DNA virus, RNA virus, enveloped virus, nonenveloped virus, cell biology, virus entry, cytoskeleton, infection, receptor, internalization, innate immunity, virion uncoating, endocytosis, gene expression, gene therapy, actin, kinesin, dynein, myosin, nuclear pore complex, adenovirus, herpesvirus, herpes simplex virus, influenza virus, hepatitis B virus, baculovirus, human immunodeficiency virus HIV, parvovirus, adeno-associated virus AAV, simian virus 40

## Abstract

Viruses have a dual nature: particles are “passive substances” lacking chemical energy transformation, whereas infected cells are “active substances” turning-over energy. How passive viral substances convert to active substances, comprising viral replication and assembly compartments has been of intense interest to virologists, cell and molecular biologists and immunologists. Infection starts with virus entry into a susceptible cell and delivers the viral genome to the replication site. This is a multi-step process, and involves the cytoskeleton and associated motor proteins. Likewise, the egress of progeny virus particles from the replication site to the extracellular space is enhanced by the cytoskeleton and associated motor proteins. This overcomes the limitation of thermal diffusion, and transports virions and virion components, often in association with cellular organelles. This review explores how the analysis of viral trajectories informs about mechanisms of infection. We discuss the methodology enabling researchers to visualize single virions in cells by fluorescence imaging and tracking. Virus visualization and tracking are increasingly enhanced by computational analyses of virus trajectories as well as in silico modeling. Combined approaches reveal previously unrecognized features of virus-infected cells. Using select examples of complementary methodology, we highlight the role of actin filaments and microtubules, and their associated motors in virus infections. In-depth studies of single virion dynamics at high temporal and spatial resolutions thereby provide deep insight into virus infection processes, and are a basis for uncovering underlying mechanisms of how cells function.

## 1. Introduction

Viruses affect all forms of life, from bacteria to humans. They are a product of co-evolution with their hosts, and cause disease, or assist in gene and anti-microbial therapies [1,2,3,4]. Virus particles, virions, require the assistance from the host cells to cause an infection, and transfer viral genes into host cells. Infection is a complex subversion process, which gives rise to latent, persistent or lytic outcomes, and cell survival or death [5]. Virions are a container with structural proteins and DNA or RNA genomes inside, sometimes wrapped with a lipid membrane and sugars. Although virions emerge from cells, their water content is several fold lower than that of cells [6]. This implies that they are tightly packaged, and contain entropic pressure [7,8,9]. Virions are considerably smaller than cells, although some of them can reach the size of bacterial cells [10]. Despite their simplicity, virus particles from different families exhibit a large structural diversity, and particles from a single virus type can contain genomes that are variable in sequence but preserve overall function. Viral genomes encode enzymes for virus replication, maturation, genome integration into the host chromosomes, as well as structural and regulatory proteins for building virions and tuning the immune system, proliferation and apoptosis.

**Figure 1:**
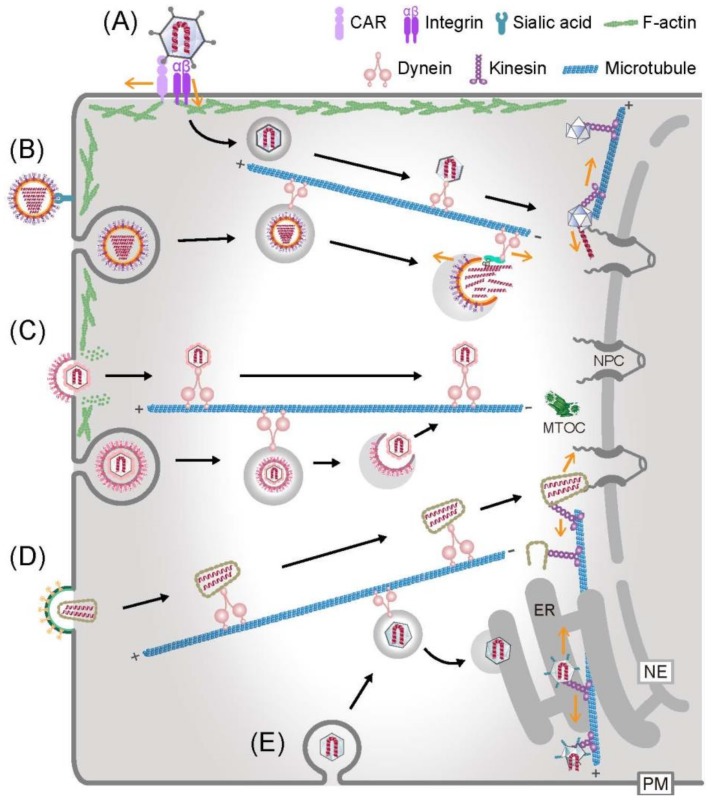
Examples of virus entry and interactions with the cytoskeleton with a focus on microtubules. Adenovirus (A), influenza virus (B), herpesvirus (C), human immunodeficiency virus (D) and simian virus 40 (E) enter into the cytoplasm either by a direct fusion of viral membrane and host plasma membrane (PM), or by receptor-mediated endocytosis, endosome rupture, or endoplasmic reticulum (ER) membrane penetration. Subsequently, viruses engage with the cytoskeleton and motor proteins to move towards the replication sites. Mechanical forces from the virus–motor protein interactions and opposing forces, such as actin-anchored integrins (A), the nuclear pore complex (NPC) (A), reverse transcription in the viral particle (D) or the site of ER penetration (E) are thought to facilitate virion disruption and release the viral genome (dark yellow arrows).

Virions deliver their genome into host cells by using receptors, attachment factors and facilitators of the host mediating binding to and activation of cells [11]. Cell signalling, endocytic uptake, endosomal escape and cytoplasmic transport all directly or indirectly depend on the actin or microtubule cytoskeleton [12,13,14,15,16,17,18,19,20,21,22,23,24]. For an overview of virus entry pathways by the cytoskeleton, see Figure 1.

Before a viral genome is transcribed and replicated, it is at least partially uncoated from the capsid. Genome uncoating requires a series of sequential interactions of the virion with host factors. This concept was initially demonstrated with adenovirus (AdV), a non-enveloped DNA virus, which starts its uncoating program by shedding the fiber proteins at the cell surface, and continues releasing minor virion components in a stepwise manner [25,26,27,28]. For some viruses, such as influenza virus (IV) and AdV, complete genome uncoating requires the acto-myosin and microtubule cytoskeleton [11,29,30]. Other viruses, such as human immunodeficiency virus (HIV) or poxviruses transcribe their genome while located in the cytosol and at least partly wrapped by their capsid [31,32,33,34]. This strategy is thought to provide protection to the viral genome from innate sensors in the cytoplasm [35,36,37,38].

Viruses replicating in the nucleus typically dissociate their genome from the capsid before the genome enters the nucleus, although very small virions, such as adeno-associated viruses are thought to uncoat their genome in the nucleoplasm [39,40,41,42,43,44]. From the site of replication in the cytosol or the nuclear compartment, newly assembled particles then leave the infected cell by mechanisms that unanimously require the assistance of the host cell [45,46,47,48,49]. This is essential because diffusion purely depends on the thermal energy, and does not suffice for effective transport of particles larger than 50 nm across the crowded cytosol [50,51,52,53,54]. In this review we discuss how the imaging of virion dynamics and trafficking in entry and egress can be analyzed by fluorescence microscopy, single particle tracking, and trajectory analyses, and thereby shed new light on cell function in health and disease.

## 2. In the Extracellular Milieu—Tracking and Modelling of Fluorescent Animal Virus Particles

The classical approach to track the motility of virions has been by fluorescence microscopy of fluorophore-tagged particles [14,52,55,56,57,58,59,60]. A list of fluorescent virus particles from both non-enveloped and enveloped families, and the methods by which the virions were prepared is presented in Table 1. The list also indicates the cytoskeletal elements and the compartments highlighted by virion tracking experiments.

Imaging studies revealed that before virions attach to cells, the particles diffuse in the extracellular medium. The cell-free medium differs drastically from the intracellular environment in molecular crowdedness [123], ion composition [124], and bulk currents [125,126]. Virus particles in the extracellular medium are subjected to advection due to thermal flow caused by temperature differences, due to the active flow of mucus caused by ciliary motions [127], or the flow of blood or lymph fluids.

Apart from the bulk currents, diffusion represents an important driving force carrying viral particles in the extracellular milieu, and contributes to virus spread in micropopulations of cells [126]. For example, the diffusion constant of particles smaller than 100 nm, such as HPV is largely unaffected by the viscosity in mucus, whereas larger particles, such as herpes simplex virus are strongly affected by mucus [127]. Similar results were reported with label-free virus particles by ultrahigh-speed scattering-based imaging, where vaccinia virus (VV) particles moved on the plasma membrane of host cells with diffusion coefficients in the range of 1 μm^2^/s [128]. Along the same lines, the diffusion constants (D) of AdV particles (approx. 90 nm in diameter) in extracellular medium are in the range of about 10 µm^2^/s, as determined by ultrahigh-speed total internal reflection fluorescence (TIRF) microscopy and single-particle tracking of fluorophore-tagged AdV-C2 using spherical fluorescent beads of different diameters as standards [125]. The results are in good agreement with the prediction of the diffusion constant (D) from the Einstein–Stokes equation, where D inversely depends on the diameter of the particle and the viscosity of the medium. Remarkably, numerous approaches have been employed towards biophysical modelling cell-free spread of viruses, including ordinary and partial differential equations (ODE and PDE) [129], agent-based models [130], cellular automata (CA), and not least multi-modal approaches combining two or more modelling methods [125,126,131]. ODE- and PDE-based approaches are among the most simplistic ways to represent dynamic changes of one or multiple parameters in a biophysical system.

## 3. The Way in and out—Actin-Based Virion Transport

After passing through the extracellular medium, the next step in the infection process is the attachment to a target cell. Eukaryotic cells lacking a cell wall, such as vertebrate cells, are shaped by a layer of actin filaments (F-actin), which also serves to reinforce the plasma membrane and imposes a barrier towards the outside of the cell. The F-actin layer is dynamically regulated by polymerisation and depolymerisation reactions, for example allowing the formation of endocytic pits and vesicles [132]. It provides opportunities for virions to bind to cell extensions, such as filopodia [14,60]. F-actin also provides contractile tracks for myosin motor proteins, and can be arranged into net-like patterns by crosslinking proteins [133]. Upon attachment to the cell surface, virions transmit forward signals into the cell, for example triggering signal transduction pathways akin to growth factors, and prepare the cell for endocytic uptake and infection [13,15,134].

The tracking of fluorophore-tagged single virions in cell culture has revealed that upon initial contacts with attachment factors and receptors on the plasma membrane, virions move outside of the cell in quasi two-dimensional diffusive motions for several seconds until their motion gets confined to small areas of a few hundred nanometers in diameter (reviewed in [14,60]). Virions remain highly mobile by engaging with the cytoskeleton through transmembrane receptors and intracellular adaptors, a process termed “surfing” (reviewed in [14,60]). Notably, fluorophore-tagged retroviruses, such as HIV, murine leukemia virus (MLV) or avian leukosis virus (ALV) [87], murine polyomavirus [107], HPV16 [79] and AdV-C2 [27] were shown to drift along filopodia, a process which is coupled to the myosin-dependent flow of filamentous (F)-actin towards the cell body where endocytic uptake processes occur. Retrograde F-actin flow was originally shown for growth cones of Aplysia neurons [135,136].

Other actin-dependent processes are subverted by viruses during entry, assembly and egress from the infected cell (reviewed in [137,138]). They include actin polymerisation-mediated movement of incoming baculovirus across the cytosol to where the virions eventually reach the cell nucleus [139], the involvement of dynamic F-actin in the entry of Influenza A virus (IAV) into polarized epithelial cells [140], herpes simplex virus (HSV) type 1 entry into cells [141], or a rapid actin-dependent intracellular movement of poliovirus and VV particles [82,116,142,143,144,145]

On the way out, actin-driven viral egress is well described for VV. The cell-associated enveloped particle induces the formation of an actin tail, and moves at an average speed of 2.8 µm/min [143,146]. This propelling force may aid in the transmission of virus particles between cells [144]. Actin-dependent transport of nucleocapsids for virion budding at the plasma membrane has also been reported for baculovirus and filovirus [139,147,148]. At the plasma membrane, F-actin interacts directly or indirectly with the matrix protein of filoviruses to drive the budding of virions, possibly involving myosin motors [149,150].

## 4. Subcellular Regulation of Microtubule-Dependent Virion Transport on the Way in and out

Microtubules are polarized filaments formed by tubulins and microtubule-associated proteins. In many eukaryotic cell types, the minus-ends are located near the cell center and plus-ends point to the plasma membrane [151]. In addition to maintaining the structure of the cells and providing the framework for cell division, microtubules serve as tracks for the intracellular transport of organelles, proteins, and RNA-protein complexes [152]. Microtubules support long range virion transport at µm/s speed. The role of microtubules in virus entry and egress from infected cells has been extensively reviewed [46,51,52,57,137,138,153,154,155,156,157,158,159]. Microtubules are composed of different isotypes of alpha and beta tubulin, which are subject to a range of post-translational modifications (PTM) [160,161,162]. PTM make up the “tubulin code”. The specific chemistry of the “tubulin code” facilitates the functional diversification, and enables interactions of subsets of microtubules with specific sets of microtubule-associated proteins, including motor proteins.

Increasing evidence indicates that viruses use and modify the tubulin code by signalling to microtubules and affecting microtubule stability and motor protein preference. On the way in, herpesvirus and HIV engage with microtubules and promote microtubule stabilization through a series of protein–protein interactions involving end-binding protein 1 (EB1) and other plus-end tracking proteins (+TIP), such as cytoplasmic linker protein 170 (CLIP-170) and kinesin family member 4 (Kif4) [163,164,165]. AdV induces microtubule growth via the Rac1 signalling pathway, and may therefore increase the chance of the formation of stable microtubules and virion engagement with these tracks [166]. Kaposi’s sarcoma-associated herpesvirus (KSHV) and HIV promote the stabilization of microtubules through the ezrin–radixin–moesin family proteins [167,168].

On the way out, IV, AdV, HIV and human herpesvirus 8 enhance the acetylation and the stability of microtubules [166,167,169,170]. In the case of IV, tubulin acetylation appears to directly promote the transport of progeny viral genomes towards the budding site at the apical plasma membrane. Yet other viruses, such as VV or HSV1, produce viral proteins with MAP-like activities to stabilize microtubules [171,172]. The HSV1 protein Us3 mimics Akt to activate cytoplasmic linker-associated proteins, which are plus-end tracking proteins (+TIP) that lead to the formation of stable microtubules [173]. HIV stabilizes microtubules by engaging the host protein suppressor of cytokine signaling 1 (SOCS1) and viral Gag protein [174]. These examples show that both incoming and outgoing virus particles stabilze microtubule tracks by PTM or dedicated viral proteins to enhance their entry or egress processes.

## 5. Virion Dismantling by the Cytoskeleton on the Way in

Viruses are too large to efficiently diffuse through the crowded cytoplasm [175]. They evolved mechanisms to take advantage of cellular transport processes, which not only affect their subcellular localization but also exert force on the particles. This has been discovered with AdV, which uses mechanical cues from motile coxsackievirus AdV receptors (CAR) and stationary integrin co-receptors on the plasma membrane of epithelial cells to shed the fibers and open-up the capsid to release the membrane lytic protein VI [9,25,26,27,30,176,177]. Virion motility on the plasma membrane depends on CAR, actin turnover and myosin-2 activity, and works against the holding force of either nonmotile CAR attached to other fibers on the virion or integrins, which are stationary. How the AdV particle exposes its membrane lytic protein in macrophages which lack CAR but provide the entry receptor SR-A6 (MARCO) is currently unknown [178]. Upon virion endocytosis and endosomal escape where the membrane lytic viral protein VI is fully exposed from the virion, the partially dismantled capsids are transported to the nuclear envelope in a microtubule-dependent manner [63,179,180].

Another important question has remained unresolved until recently, namely how any virus particle, which traffics on microtubules can detach from microtubules in order to bind to the nuclear pore complex (NPC) in the nuclear envelope. This question is of key importance for herpes viruses, HIV, parvoviruses, IV viral ribonucleo-protein complexes (vRNP), human foamy virus and AdV, which all deliver their genome into the nucleus of postmitotic cells [52]. Mechanisms of cellular cargo unloading from microtubules vary depending on the types of motors and cargos [181,182]. In the case of AdV, it was recently shown that the nuclear export factor CRM1 has a key role in controlling the virus–microtubule interactions at the juxtanuclear region [183]. Upon the inhibition of CRM1 by leptomycin B, the incoming AdV is constrained on the microtubules, and fails to reach the NPC. If the virions are allowed to detach from microtubules in the juxta-nuclear region, they attach to the NPC via a hexon-Nup214 interaction [183,184,185]. At the NPC, the molecular motor kinesin-1, which is tethered to the virion is activated by Nup358, and then exerts a mechanical force, which breaks open the weakened capsid and releases the viral genome for import into the nucleus through the NPC [29].

The mechanical uncoating concepts proposed for AdV have been adapted for IAV entry [186]. In this scenario, the vRNPs are dissociated from the endosomal membrane by dynein and myosin motors, upon exposure to the cytosol and fusion of the viral membrane with the limiting endosomal membrane. Along a similar line, conventional kinesin has been implicated in membrane penetration of SV40 particles from the ER lumen to the cytosol [187]. Membrane penetration depended on acetylated microtubules in agreement with the earlier notion that kinesin-1 preferably moved on acetylated microtubules [188,189]. In addition, the destabilization of the HIV capsid was reported to depend on microtubules and dynein motor activity [190]. In summary, it becomes increasingly clear that actin filaments, microtubules and associated motors serve as cues for scheduled on-site virus capsid disassembly events, and thereby boost viral infection.

## 6. Tool Box—Virion Imaging in Cells

Fluorescence microscopy has empowered virologists to determine how virions interact with cell surface attachment factors, receptors, and facilitators for cell entry [11,13,14,56,57,71,105,178,191]. Imaging post-entry steps has shown how the cytoskeleton and its motors support virion trafficking and uncoating, leading to gene delivery and infection [52,138,155,156,157,192,193]. Here we provide an overview of imaging modalities by light microscopy with viruses (Table 2).

Diffraction-limited light microscopy has a spatial resolution in the range of several hundred nanometers, as described by Abbé’s law [220]. Most viruses are between 20–400 nm in size, below the diffraction limit, and appear as point sources in micrographs [221]. However, at high fluorescence signal from the particles and low background one can use the point spread function to determine the position of the particle with much higher precision than the resolution of the microscope. Single particle tracking at high signal to noise ratio is thus possible at low tens of nanometer accuracy, and is providing unprecedented information (see also below). For example, particles that have been derivatized with organic fluorophores or genetically fused to a fluorescent protein, such as GFP could be tracked below the diffraction limit of the light microscope [222]. Alternatively, fluorescence can be induced on the virion by the so-called split-GFP technology, where an engineered particle contains a GFP segment, and binds to a protein of interest with another GFP segment in presence of soluble GFP core, which together leads to complementation of fluorescence on the virion [223]. This three-way complementation informs about virion proximity to cytoplasmic proteins of interest.

Fluorescent AdV particles were shown early on to use the microtubule-dependent transport system to traffic to the nucleus and uncoat the genome at the nuclear pore complex [29,63,224]. Fluorophore-tagged SV40 revealed actin-dependent virus entry [225], and fluorophore-tagged human papillomavirus (HPV) 31 pseudovirions were tracked by confocal fluorescence microscopy to reveal the movement of single virus particles via retrograde transport from the filopodium periphery toward the cell body [80,226]. Experiments with quantum-dot labeled IAV particles suggested a motor switch on the endosome from myosin VI to cytoplasmic dynein [227], and GFP-tagged HSV1 capsids were found to traffic on microtubules [228,229,230,231]. In addition, the bis-arsenical fluorescein derivative FlAsH reveal trafficking of HIV particles [232]. For IAV, organic fluorophore labeled isolated vRNPs that were microinjected into cells revealed cytoplasmic trafficking [233], whereas fluorophore-tagged IAV particles that were endocytosed into cells highlighted the impact of the microtubule network on endosomal transport processes [104]. A summary of fluorescent virus particles has been provided in Table 1.

The recent development of superresolution microscopy, including single molecule localisation microscopy (SMLM) [204,205], stimulated emission depletion microscopy (STED) [202], and structured illumination microscopy (SIM) [234], has been breaking the diffraction-limited resolution barrier. However, most superresolution microscopy methods have a low acquisition speed, high phototoxicity and require heavy post-acquisition processing, which makes volumetric time-lapse imaging of live cells impractical [235]. Nonetheless, superresolution fluorescence microscopy has been used to gain subcellular localization information of virions and viral genomes in chemically fixed cells with single particle resolution [14,52,56,57,66,183,236]. This has demonstrated, for example, that cytosolic AdV particles are leaky containers that have undergone limited disassembly, yet still perfectly enclose the viral genome and shield it against cytosolic sensors and innate immunity [28,65,66].

In addition to fluorescence microscopy, label-free microscopy based on interferometry has been used to track the motions of extracellular vesicles and HSV1 particles, as well as SV40 particles on supported membrane bilayers harbouring the SV40 glycolipid receptor [215,237]. Optical interferometry is a technique which records an interference pattern of superimposing electromagnetic waves following interaction with an object. Interferometry is a low-invasive approach to reveal the dynamics of virion trafficking at high temporal and spatial resolution.

Another emerging development to unravel molecular details of virus–host interactions is force spectroscopy applied to single virions, including atomic force microscopy (AFM) and optical tweezers (OT) [238]. This allows for the mapping of physical properties of virions and the impact of particle physics on the entry of HIV, HSV1 and AdV into cells [8,9,239,240,241,242,243,244]. AFM and OT have also been used for determining force–distance curves in virion binding to cells using microscale thermophoresis, and for mapping of the interactions of virion components with potential cell surface adhesion molecules [238,245].

Another label-free imaging modality to track virions was recently introduced, coherent brightfield microscopy (COBRIM). COBRIM detects scattered light by imaging-based interferometry. It can be combined with digital image processing and post-processing to remove background scatter noise from cellular structures, and thereby allowed to track the motions of VV particle at nanometer accuracy and microsecond temporal resolution [128]. 

## 7. Tool Box—Single Virus Particle Tracking

**Figure 2:**
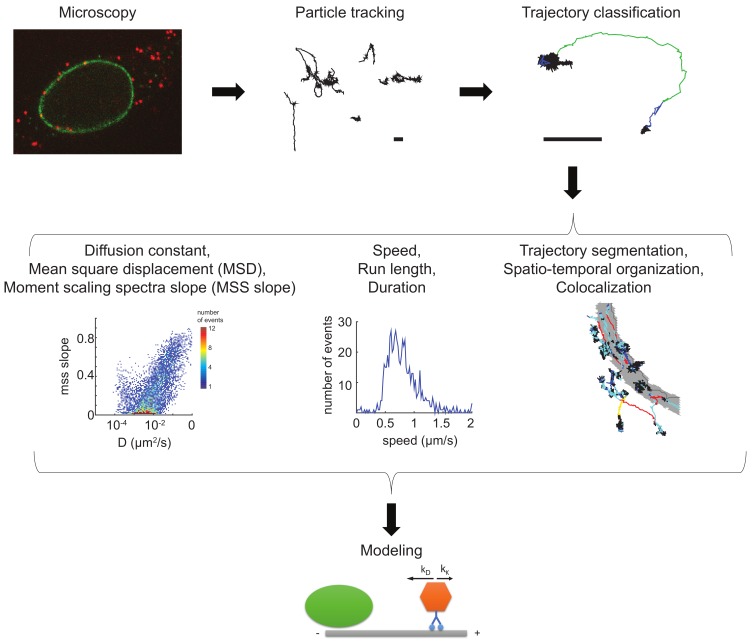
Workflow of a virus imaging and single particle tracking experiment. Images from fluorescent viral particles infecting host cells are acquired at high temporal and spatial resolution (microscopy). Images are processed and particles detected and tracked over time (particle tracking). The resulting virus trajectories can be analyzed by a plethora of different and orthogonal approaches, including diffusion and moment scaling spectrum slope measurements, and trajectory segmentation (schematic depiction in the center row). Motion properties can be extracted from entire tracks or from segments. Virus particles in motion can contain fluorescent cellular marker proteins or localize with subcellular compartments. Parameters extracted from the motion behavior can be used for various biocomputational modeling approaches to generate predictions that can be tested in follow-up experiments (modeling). Scale bars: 1 µm. The segmented tracks on grey shaded nuclear outlines of HeLa cells have been derived from previously published data, and were adapted with permission from The Company of Biologists Ltd. (Cambridge, UK) [183].

Initially, virion motions were manually tracked with limited precision and small numbers of trajectories [62]. Advances in virion labelling, microscopy engineering and image analyses have recently allowed for live-cell observation of thousands of virus particles at high temporal and spatial resolution. Single particle tracking experiments are analyzed in three distinct steps: particle detection and tracking, trajectory classification, and physical modeling [200]. A typical workflow of a virion imaging and tracking experiment is depicted in Figure 2.

Below, we highlight recent applications in virus imaging and single particle tracking, and discuss advances and challenges. Viral particles are detected in time-lapse image series, time-resolved particle positions are recorded, and the resulting trajectories are analysed and particle motion is classified (reviewed in [196,246,247,248]). Imaging modalities used for virus tracking range from wide field microscopy, total internal reflection microscopy (TIRF) to ring sheet light-sheet microscopy and confocal microscopy. All these techniques allow for the detection of virions labelled with dozens of photostable small organic fluorescent dyes or fluorescent proteins at high signal to noise ratios [210,249]. The strong signals emitted from the diffraction limited viral particles allow for highly accurate particle localization in the nanometer range [249], well below the resolution of the microscopes.

For dim signals from particles labelled with only few copies of GFP-fusion proteins [65], or for signals from rapidly moving particles [180,250], the detection of particles has been challenging. Rapidly moving particles cover a longer distance during the acquisition time of the image and depending on the frame rate in image acquisition, their signal may spread over a large area and lose peak intensity. As the signal degenerates, the particles become harder to detect and localization becomes less accurate. This has been noticed, for example, by advanced wavelet-based transforms, which are mathematical operations to characterize spatial image information over large space scales with greater accuracy than intensity-based segmentation algorithms. More recent developments have replaced the wavelet-based transforms by model-based detection algorithms, which score low signal to noise image data [251], and improve the detection of dim diffraction limited objects, such as clathrin-coated pits [252].

A variety of tracking algorithms are available for diffraction-limited objects, such as virus particles. The performance of trackers was assessed in an unbiased “grand-challenge” on synthetic and real data, including virus motion [253]. The goal for research groups was to independently apply their algorithms on a set of test cases, and to compare the results based on common evaluation criteria. Performance varied depending on the task, and certain trackers performed particularly well on the virus motion data [249,254,255].

Nonetheless, tracking of heterogeneous and crowded motions of virions on the cell surface or in the cytoplasm remains challenging, especially if the particles appear and disappear from the focal plane of observation. Particle appearance/disappearance can be due to the curvilinear rails of microtubules serving as tracks for high speed virion motions [52]. To computationally connect discontinuous tracks, gap closing algorithms have been successfully applied post-processing [251].

**Figure 3:**
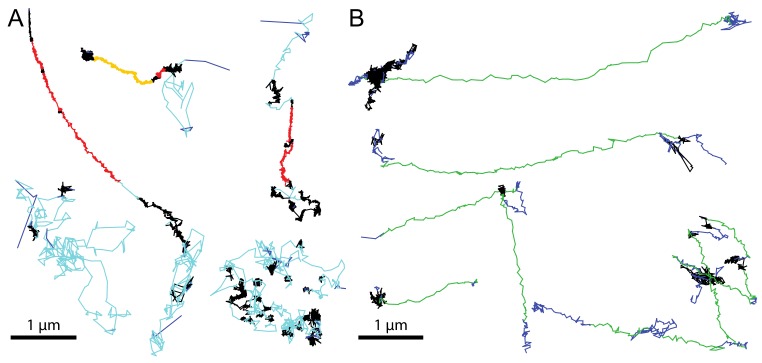
Examples of virion trajectories on the cell surface and in the cytosol. Segmented trajectories from AdV at the cell surface (**A**) and in the cytoplasm (**B**). Cell surface motion was classified in diffusion (cyan), slow drift (red), fast drift (orange), confined motion (black) and not classified steps (blue). Cytoplasmic motion was classified into directed motion (green), fast and slow drifts (orange and red, respectively), confined motion (black) and not classified steps (blue). Scale bars are 1 µm. Technical details of the tracking and segmentation procedures are described in [27]. The segmented tracks in panel A were derived from previously published data, and adapted with permission from Elsevier [27]. The segmented tracks in panel B were adapted from [183], with permission from The Company of Biologists Ltd.

Molecular crowding occurs, for example, when virions are tethered to microtubule or actin filaments at high filament density. Heterogeneous virion motions exhibit rapidly changing directionality, stop-and-go bursts, long periods of spatial confinement or fast transitions between motion types [200]. To improve the tracking in crowded conditions, a piecewise-stationary motion model smoother (PMMS) approach was developed and tested on the cytoplasmic motion of AdV particles [256]. PMMS applies an iterative recursive tracking approach, where particles are tracked in both forward and backward direction over multiple iterations. This helps recovering tracks that undergo spontaneous transitions in motion patters [256], for example between confined and directed motion, as it is frequently observed for cytoplasmic motion of AdV (see Figure 3). Significantly, the advent of advanced light sheet microscopy will enhance further developments in tracking of diffraction limited objects in 3D [257,258,259].

## 8. Tool Box—Trajectory Analyses

Various classification methods for single particle trajectories have been proposed. Significantly, all the microscopy software packages for object tracking contain a trajectory classifier [247,260,261]. Table 3 provides a compilation of different tracking and trajectory analysis software used for viruses.

The most commonly used trajectory classification describes the speeds of the entire tracks, or domains within heterogeneous tracks. For example, the reported transport speeds of virions on microtubules covered a wide range of speeds for different viruses, with top speeds in the range of several µm/s (Table 4).

**Figure 4:**
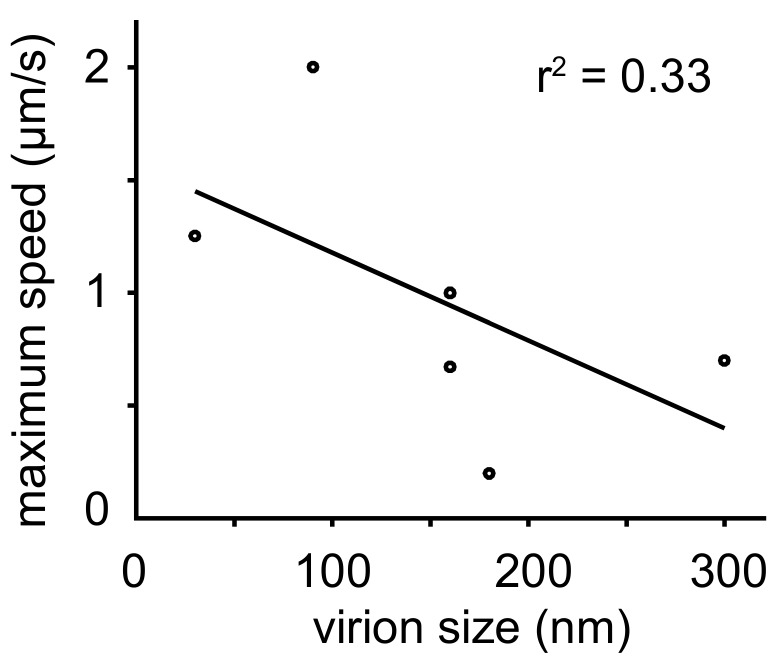
Plot of virion speed on microtubules versus particle size. Microtubule-based speed of viral particles inversely correlates with particle size. Reported maximum speeds and particle sizes are shown. For details and references, see Table 4.

A meta-analysis revealed an inverse correlation between particle size and transport speed (see Figure 4). Small viruses like FMDV (25–30 nm) and AdV (90 nm) moved at maximum speeds of 1–2 µm/s, while much larger rabies virus (180 nm) and VV intracellular enveloped particle (IEV) (300 nm) were transported at speeds around 0.5 µm/s. Speed reduction could be explained by increased drag forces on particles with larger radii in agreement with the Stokes law [268]. Larger particles may also be slowed down in a crowded cytoplasm by entanglements with organelles and filaments. Alternatively, larger viruses may offer more binding sites for motor proteins of opposite directionality and hence may get slowed down by tug-of-war [250].

In addition to the overall particle velocity, the peak speeds and continuous drifting speeds, the mean square displacement (MSD), diffusion coefficients, the moment scaling spectra slopes (SMSS), and segmentations have been widely used for characterization and classification of viral trajectories [269,270]. A compilation of motion features that have been extracted by virus trajectory analyses is provided in Table 5.

In fact, some of the first virus tracking experiments were reported with fluorescent IV particles on the surface of human fibroblast in cold medium [103,271]. MSD analyses indicated that the particles underwent random diffusion and continuous drifts, albeit at very low frequency. The slope of the momentum scaling spectrum classifies the motion diffusivity into confined, diffusive and superdiffusive motions [249,272]. SMSS analysis is useful and powerful for moving object analysis [273], and especially for viruses, since virus trajectories are heterogeneous and rarely highly processive due to virion interactions with a range of host factors, as amply demonstrated by cell surface motion analyses of AdV, for example [27]. In addition, SMSS analysis identified filopodial drifting motions of murine poliomavirus virus-like particles and HPV16 pseudoviruses, which are assembled from recombinant capsid proteins and lack a viral genome [79,196].

Heterogeneous motion behavior is readily revealed by imaging at high spatial and temporal resolution [274]. For example, transient confinement zones have been investigated for a variety of molecules [275]. Algorithms have been developed to detect jumps between adjacent confinement corrals and sudden changes in diffusion coefficients [276,277,278] or SMSS for intracellular Mason–Pfizer Monkey retroviral (M-PMV) particles [261].

Differential motion behavior was also detected by moving window approaches, where motion parameters are extracted from sub-segments and changes in motion behavior are defined by thresholding methods [276,279] or segment classification [280]. In addition, confinement zones were detected using Bayesian methods [281,282], Hidden Markov models [283] and particle filtering methods [284]. Likewise, Hidden Markov models were used to extract dynamic colocalization events from multi-channel image data in the case of HIV membrane fusion events [285].

Importantly, machine learning approaches were introduced for trajectory analysis including support vector machines (SVM) for AdV motion analyses [200]. Virion trajectories were segmented into different motion modes including confined motion, directed motion and drifts with supervised SVM classification [200] (Figure 4). Subsequently, neural networks were developed for the analysis of membrane receptor motion [286]. While single particle tracking algorithms converged to more robust solutions in the past decade [253], novel motion analysis procedures are being developed. This will likely enhance the analyses of complex and heterogeneous motion behaviors in 2D and 3D biological samples, and multi-channel data sets. Interference approaches and analyses of complex virus motions are expected to give insight into biological mechanisms of host cell and tissue infections in the course of disease.

## 9. Tool Box—Physical Models Describing the Movements of Incoming Virus Particles

Computational modeling and simulations can provide in silico experimental data to enhance mechanisms and give insight into interaction processes. Increasingly, aspects of infection dynamics are addressed by mathematical and computational modeling (for reviews, see [287,288,289]). Parameters extracted from virus tracking experiments can be used in data-driven models which are also known as “top-down” models. They explore patterns and make correlations from statistical or machine learning methods. Examples include correlation analyses, reconstruction of molecular interaction networks from high-content screening datasets, and classification of viral motion types using machine learning [200,290,291]. Data-driven models suggest mechanisms, and can indicate necessity of a process or a molecule in a perturbation experiment [289]. This is notably different, yet complimentary to so called “bottom-up” modelling, where physical models are constructed based on mechanistic hypothesis derived from prior data or theoretical reasoning [289]. In contrast, the “top-down” approach allows for discovery of new patterns concealed in the data.

With regard to cytoskeletal transport, bidirectional active transport along microtubules, propelled by dynein and kinesin motors has been an attractive subject for modelling studies. A general mathematical model for stop-and-go virus motion on microtubules was proposed [287,292,293]. In addition, a stochastic computational model of AdV cytoplasmic transport was built using parameters extracted from single particle tracking and trajectory segmentation experiments [250]. This model allowed the possibility of a tug-of-war between motors of opposite migration on microtubules, such as cytoplasmic dynein and conventional kinesin [294]. The model also allowed predictions of the number of active motors attached to single virions during fast directed motions, namely two to three, and the number of the virion binding sites for the microtubule motors, which implied the major virion capsid protein hexon [250]. Subsequently, empirical evidence confirmed that hexon directly binds to the microtubule minus-end trafficking motor protein, the dynein complex [64].

## 10. Conclusions and Outlook

An increasingly refined picture is emerging of how virus particles traffic on the cytoskeleton during entry and egress from cells. The knowledge emerges from analyses of virion trajectories by computational methods, and in silico simulation experiments. Advances in virion imaging and motion analyses provide a basis to inform about biological mechanisms, and foster the development of anti-viral therapeutics. They critically enhance the conceptual understanding of cell functions, based on the notion that viruses interact with thousands of proteins in an infected cell, and are major drivers of host adaption in evolution, and immune regulation.

Open questions and challenges comprise virus trafficking studies in cultured cells, primary cells and tissues, and the complex communication processes of the host to and from the pathogen. For example, the deciphering of how cytokines, chemokines and other signalling molecules affect the way viruses use the cytoskeleton and tune cytoskeletal dynamics will inform passive and active pro- and anti-viral mechanisms. Deep analyses of virion motions in cells will further inform on underlying mechanisms of cell-to-cell heterogeneity of infection, a phenomenon which is largely unexplored but well known to every virologist. Finally, time-controlled virus infection analyses will better prepare the field to reach out to even more complex settings, for example the elucidation of the interactions between eukaryotes, microbes and viruses in the course of human health and disease.

## Figures and Tables

**Table 1:** Fluorescent animal viruses.

Virus	Virus Family	Labeling Method	Cytoskeleton/Compartment	References
*Non-enveloped*				
Adeno associated virus (AAV)	*Parvoviridae*	Quantum dots, GFP-VP2	Cell surface, endosomes, nuclear import	[61]
Adenovirus type 2, type 5 (AdV-C2,5)	*Adenoviridae*	Small organic dyes (Cy5, TexasRed) GFP-pV Ethynyl-modified nucleosides	Microtubules, plasma membrane, actin, clathrin-coated pits, nuclear pore complexes	[62,63,64,65,66,67,68,69,70,71,72,73]
Adenovirus type 35 (AdV-B35)	*Adenoviridae*	Small organic dyes (TexasRed)	Macropinosomes, actin ruffles	[73]
Canine Parvovirus	*Parvoviridae*	Small organic dye (Cy3, AlexaFluor)	Clathrin-coated pits	[74,75]
Echovirus-1 (EV-1)	*Picornaviridae*	Small organic dye (AlexaFluor 594)	Clathrin-coated pits, Trans-Golgi-Network (TGN)	[76]
Foot and mouth disease virus (FMDV)	*Picornaviridae*	Small organic dye (AlexaFluor 555)	Microtubules	[77]
Human papillomavirus (HPV) 16 HPV16-Pseudo-virions, Human papillomavirus 31 (HPV31)	*Papilloma-viridae*	Small organic dyes (FITC, AlexaFluor 488, 594)	Filopodia, plasma membrane, actin	[78,79,80]
Poliovirus serotype 1 (PV1)	*Picornaviridae*	RNA binding dye (Syto82), small organic dye for capsid labeling (Cy5)	Actin, microtubules	[81,82]
Reovirus	*Reoviridae*	Small organic dye (AlexaFluor 647)	Clathrin-coated pits	[83]
Rhinovirus (RV)	*Picornaviridae*	Small organic dye (FITC)	Plasma membrane	[84]
Simian virus 40 (SV40)	*Polyomaviridae*	Small organic dye (TexasRed)	Plasma membrane, caveolae, actin comets	[85]
*Enveloped*				
African swine fever virus like nanoparticles (ASFV)	*Asfarviridae*	p54 peptide aa149-161 tagged liposomes	Microtubules	[86]
Avian leucosis virus (ALV)	*Retroviridae*	MLV Gag-CFP, pseudotyped with ALV EnvA	Filopodia, plasma membrane, actin, cytonemes	[87]
Chikungunya strain LS3-226A	*Togaviridae*	Lipophilic DiD dye	Membrane, clathrin, early endosomes	[88]
Dengue Virus serotype 2 (DENV2)	*Flaviviridae*	Lipophilic dye DiD	Clathrin-coated pits	[89]
Ebolavirus GP EboV pseudotyping VSV	*Filoviridae*	Lipophilic dye DiL AlexaFluor 647-VSV-GP-EboV	Macropinosomes, actin endosomes	[90,91]
Feline Coronavirus (FCOV)	*Coronaviridae*	Virus interior labeled with Sulforhodamine B (SRB), Virus membrane labeling with lipophilic dye Rhodamine 110 C18 (R110C18)	Membrane fusion with lipid bilayers	[92,93]
Herpes Simplex Virus-1 (HSV1)	*Herpesviridae*	VP26-Venus, VP22-mRFP, VP13/VP14-mRFP and Venus gB-CFP Ethynyl-modified nucleosides	Golgi, Trans-Golgi-Network (TGN), nucleus microtubules	[66,94,95,96,97]
Hepatitis B virus- like particles	*Hepadnaviridae*	Hepatitis B surface antigen particle (HBsAg)	Actin	[98]
Hepatitis C virus (HCV)	*Flaviviridae*	Lipophilic dye DiD	Actin	[99]
Human Immune-deficiency virus (HIV)	*Retroviridae*	Gag-GFP GFP-VPR	Filopodia, plasma membrane, actin, cytonemes microtubules	[100,101,102]
Influenza virus A X-31 (IAV)	*Ortho-myxoviridae*	Lipophilic dye DiD, R18	Plasma membrane, clathrin-coated pits, microtubule motion	[103,104,105]
Murine leukemia virus (MLV)	*Retroviridae*	Env-YFP, Gag-CFP	Filopodia, plasma membrane, actin, cytonemes	[101,102]
Murine polyoma virus like particles (VLP)	*Polyomaviridae*	Small organic dyes (FITC, AlexaFluor 594)	Plasma membrane, actin	[102,106,107]
Rabies virus (RV) Street rabies 9 vaccine (SRV9)	*Rhabdoviridae*	VSV-G pseudotyped with rabies-G, Small organic dyes (Cy5) EGFP-P, P-EGFP	Filopodia/actin, clathrin-coated pits, microtubules	[108,109,110]
Respiratory Syncytial Virus (RSV)	*Pneumoviridae*	Nano Gold-coated viruses	Plasma membrane	[111]
Semliki Forest Virus (SFV)	*Togaviridae*	Small organic dye (FITC)	Microtubules, early and late endosomes	[112,113]
Sindbis virus (SINV)	*Togaviridae*	mCherry-E2	Virus budding from plasma membrane	[114]
Uukuniemi virus (UUKV)	*Phenuiviridae*	Small organic dyes	Cell surface, actin	[115]
Vaccinia virus (VV)	*Poxviridae*	Intracellular enveloped virus (IEV): B5R-EGFP F13L–GFP Intracellular mature virus (IMV) EGFP-A5 Ethynyl-modified nucleosides	Microtubules, actin, macropinosomes, plasma membrane, actin ruffles and membrane blebs	[66,116,117,118,119]
Vesicular stomatitis virus (VSV)	*Rhabdoviridae*	Small organic dye (Alexa Fluor 647)	Clathrin-coated pits, actin	[120,121]
West Nile Virus (WNV) subviral particles (SVPs)	*Flaviviridae*	Lentivirus pseudotyped with WNV prM-E, labeled with lipophilic dye DiD	Microtubules	[122]

**Table 2:** Techniques for live cell visualization of viruses and pros and cons in virus tracking experiments. Additional examples of techniques used for virion imaging are listed in Table 5.

Imaging Technique	Pros	Cons	Viruses/Ref.
**Wide-field microscopy:** Epi-fluorescence microscopy [194]	Ease of use and high accessibility	Out of focus light increases noise levels	Adenovirus type 2 (AdV-C2) [63]
Real time light scattering of gold nanoparticle coated viruses	High image contrast and temporal resolution	Limited availability of labels	respiratory syncytial virus (RSV) [111]
Total internal reflection microscopy (TIRFM) [195]	Illumination of a few 100nm thin layer reduces background signal	Only cover glass attached basal membrane is accessible	Murine polyoma virus like particles [196], AdV-C2 [27]
**Confocal microscopy:** Laser scanning microscopy [197] Spinning disc confocal microscopy [198,199]	Eliminated out of focus light increases contrast Good multi-dimensional spatial and temporal resolution	Phototoxicity and photobleaching, particularly for laser scanning confocal microscopy	AdV-C2 [200], Poliovirus (PV) [81], Reovirus [83]
**Super resolution imaging:** [201] Stimulated emission depletion microscopy (STED) [202] Structured illumination microscopy (SIM) [203] Stochastic optical reconstruction microscopy (STORM) [204] Photoactivated localization microscopy (PALM) [205]	Superior spatial resolution Most powerful with fixed samples	Limited availability of photoswitching labels for PALM and STORM Some fluorophores require special buffers that can affect cell viability Low time resolution limits usefulness for live cell imaging High demand on data post-processing	Rotavirus [206] VSV-like particles [207] Influenza A virus (IAV) [208]
**Light sheet microscopy:** [209] Ring sheet microscopy LS-RESOLFT	Rapid 3D imaging Low phototoxicity	Custom built microscopes require tailored sample chambers and training in instrument alignment and operation Large volumes of multidimensional data are generated. Data storage and analysis require extensive IT infrastructure	Herpes simplex virus (HSV1) [210], human immune-deficiency virus (HIV) [211]
**Scanning surface confocal microscopy**	Direct correlation between fluorescent signals and topography of cell surface Low background from autofluorescence	Low scan rate Phototoxicity and bleaching Low spatial resolution for rapidly moving objects	Polyomavirus-like particles (PyV-like) [212]
**Atomic force microscopy** [213]	High recording frequencies (>1000 Hz) Force measurement Superior spatial resolution	Potential interference by tip scanning Longer acquisition interval for imaging Difficult to scan large areas	Singapore grouper iridovirus (SGIV) [214]
**Label free interferometric confocal microscopy**	Label-independent Low phototoxicity Long observation periods and high temporal resolution	Limited depth-of-field along z-axis Limitation in multiplexed imaging and automated signal segmentation	Simian virus 40 (SV40) [215]
**Correlated light and electron microscopy**	Visualization of ultrastructural features identified by light microscopy	Currently, limited compatibility with live imaging	HIV [216], AdV [29,217,218,219]

**Table 3:** Open source tracking and trajectory analysis packages. Adapted from [253,261].

Software	Website	Features	References
Mosaic	http://mosaic.mpi-cbg.de/?q=downloads	ImageJ plugin and Matlab toolboxes	[249]
U-track	http://www.utsouthwestern.edu/labs/danuser/software/	Matlab toolbox	[251,262,263]
MotionTracking	http://motiontracking.mpi-cbg.de/get/	Windows executable	[264]
TrackMate	http://fiji.sc/TrackMate	ImageJ plugin	[265]
ICY	http://icy.bioimageanalysis.org/	Java application	[266]
OMEGA	https://github.com/OmegaProject/Omega	Java application	[261]
STAWASP	http://dx.plos.org/10.1371/journal.pone.0163437	Matlab source code and executable	[260]
Diatrack	http://diatrack.org/	Windows executable	[267]

**Table 4:** Virion speeds on microtubules.

Virus	Speed	Particle (Size, Type)	References
West Nile Virus (WNV) subviral particles (SVPs), pseudotyped lentivirus	Range: 0.012–0.67 µm/s (N = 55)	90–160 nm, enveloped	[122]
Vaccinia virus (VV) IEV	Average 0.8 ± 0.2 µm/s (N = 20) Range: 0.2–1 µm/s (N = 5) Range: 0.3–0.69 µm/s (N = 6)	200–400 nm, enveloped	[116,117,118]
Adenovirus type 2 (AdV-C2)	Directed motion range: 0.2–2 μm/s (N > 1000)	90 nm, non-enveloped	[63,180]
Foot and mouth disease virus (FMDV)	Range 0.5–1.25 µm/s (N = 10)	25–30 nm, non-enveloped	[77]
Rabies virus	Range: 0.05–0.2 µm/s (N>1000)	180 nm, enveloped	[109]
HIV	Up to 1 µm/s burst (N = 5)	90–160 nm, enveloped	[100]

**Table 5:** Trajectory analyses extract a range of virus motion features. Total internal reflection (TIRF) microscopy.

Feature	Virus	Motion Process/Compartment	Microscopy	References
*Speed*				
	Nanoparticles modified with African swine fever virus p54-derived peptide	Cytoplasmic linear transport	Epi-fluorescence	[86]
	Reovirus	Targeted motion to clathrin-coated pits	Spinning-disc confocal	[83]
	Foot and mouth disease virus	Microtubule-based motion	Epi-fluorescence	[77]
	Retroviruses	Drifts on filopodia	Confocal	[87]
	Hepatitis B virus-like particles	Directed motion in the cytoplasm	Confocal	[98]
	HIV	Motion on filopodia	Confocal	[101,102]
	Influenza A virus	Endocytosis	Epi-fluorescence	[105]
	Rabies virus	Virus internalization	Confocal	[109]
	Vaccinia virus	Intracellular linear transport	Epi-fluorescence	[116,117,118]
	West Nile virus	Intracellular transport	Epi-fluorescence	[122]
	Adenovirus (AdV-C2)	Microtubule-based cytoplasmic motion	Spinning-disc confocal	[180,183]
*Diffusion constant*				
	Dengue virus	Diffusion towards clathrin-coated pits	Epi-fluorescence	[89]
	Feline coronavirus	Diffusion on supported bilayers	TIRF	[92]
	Vesicular stomatitis virus	Plasma membrane motion, clathrin mediated endocytosis	Spinning-disc confocal	[120]
	Adenovirus (AdV-C2)	Cell surface motion	TIRF	[27]
*Mean square displacement (MSD)*				
	Influenza A virus X47	Cell surface motion	Epi-fluorescence	[103]
	Canine parvovirus	Cell surface, clathrin-coated pits	TIRF, Spinning-disc confocal	[75]
	Poliovirus	Actin-based motion	Spinning-disc confocal	[82]
	Hepatitis C virus	Endocytosis	Spinning-disc confocal	[99]
*Slope of the momentum scaling spectrum (SMSS)*				
	Murine polyoma virus–like particles (VLPs)	Cell surface diffusion and drifts on filopodia	TIRF	[107]
	HPV16 pseudovirions	Drifts on filopodia	TIRF	[79]
	Mason–Pfizer Monkey retrovirus (M-PMV)	Assembly, cytoplasmic transport		[261]
*Segmentation*				
	Adenovirus (AdV-C2)	Cell surface and microtubule-based transport	TIRF, Spinning-disc confocal	[27,180,183]
	Mason–Pfizer Monkey virus (M-PMV)	Assembly, cytoplasmic transport		[261]
